# When Does Group Efficacy Deteriorate Group Performance? Implications of Group Competency

**DOI:** 10.3390/bs12100379

**Published:** 2022-10-02

**Authors:** Haesang Park, Sooyoung Shin

**Affiliations:** 1 Heller College of Business, Roosevelt University, Chicago, IL 60605, USA; 2 School of Business, Yeungnam University, Gyeongsan 712749, Korea

**Keywords:** group composition, group competency, group efficacy

## Abstract

While the social cognitive theory suggests that a group’s efficacy belief enhances its performance, emerging evidence indicates that this relationship is more complex than it appears to be. This study explores the boundary conditions of this relationship using the data of 389 employees from 41 work groups in a manufacturing company in South Korea. The results show that group efficacy is positively related to group performance and that this relationship is stronger when members are generally incompetent than competent. We also found that a bottleneck, which is operationalized as a group’s minimum competency, in an efficacious group is at least one condition that forms a negative relationship between group efficacy and its performance.

## 1. Introduction

Self-efficacy, which refers to one’s belief in their ability to perform a task within a given context, is essential to work motivation [1] The social cognitive theory posits that individuals with a high level of self-efficacy tend to perform better than their counterparts by striving to fulfill higher task goals [2,3]. At the group level, studies have shown that group efficacy, which refers to “a group’s shared belief in its conjoint capabilities to organize and execute the courses of action required to produce given levels of attainments” [2] (p. 477), promotes group performance [2,4,5]. 

Although the positive relationship between group efficacy and performance seems straightforward, some empirical studies have revealed alternative patterns [6,7,8] and moderators that affect this relationship [4,5,9]. The complexity of the relationship partly arises from the fact that group efficacy is based on social interactions among group members and the process of collective cognition [1,9,10]. Unlike an individual member, group members naturally experience agreements or disagreements about their group and work process. Moreover, not only the group context, but also a distinctive individual member may change the interpersonal dynamics within a group and even steer the group in a different direction, yet we know only little about the boundary conditions. Building on this line of inquiry, we explore the boundary conditions in this relationship. 

**Figure 1:**
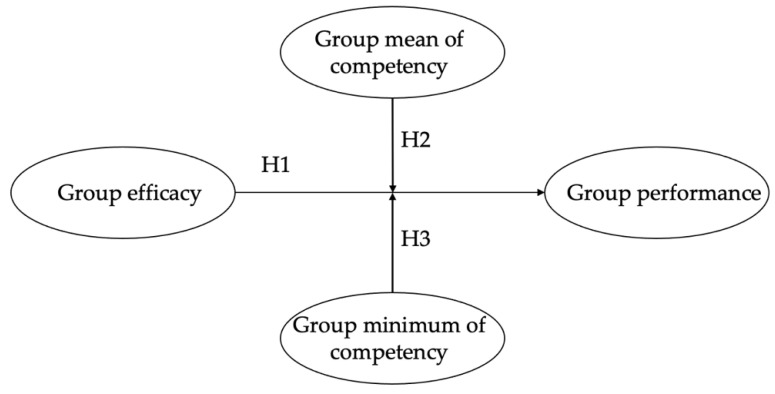
Research Model.

Competency refers to employees’ abilities and characteristics that are considered desirable in a specific organizational context [11]. An employee with a higher level of competency tends to be successful in a specific organization [12,13,14]. By applying individual-level competency to the group level, we illustrate how group competency may influence the relationship between group efficacy and group performance. Workgroups achieve their goals by coordinating the interdependent acts of individual members [15], in which group members play both productive and counterproductive roles and are naturally influenced by each other [16]. When members are generally incompetent in achieving the task goals or the group has an individual member who is perceived as a bottleneck, group performance may not develop as planned. In this study, we offer more nuanced ideas about the relationship between group efficacy and group performance by exploring two aspects of group competency. First, we examine how the group average of member competency affects this relationship. Second, we explore the role of the least competent member, who is most likely to impede the process of achieving the group’s goals. The research model is illustrated in Figure 1. 

In the following sections, we first develop hypotheses based on the existing literature and theories and examine our hypotheses with a field study. Then, we discuss the results and implications of our findings.

## 2. Theoretical Framework & Hypothesis Development

### 2.1. Group Efficacy and Performance

Based on social cognitive theory [2], researchers have suggested that self-efficacy influences performance both directly, by increasing an individual’s motivation to work, and indirectly, by encouraging an individual to pursue more difficult goals [17]. Studies have been conducted in various areas including workplace, educational settings and clinical settings. Moreover, even in difficult situations, a sense of efficacy helps to sustain motivation and overcome challenges. Empirical studies have found that self-efficacy is positively related to work-related outcomes across various domains [3,18,19]. 

Group efficacy also operates through a paradoxical process, similar to self-efficacy, and has similar consequences [2,9,20,21,22,23]. The sense of confidence formed by group efficacy motivates members [24]. Specifically, group efficacy drives members to pursue more challenging goals, expend more effort to achieve those goals, and be resilient in difficult situations [25]. Empirical studies [9,26] and meta-analyses [4,5] support the positive relationship between group efficacy and group performance.

**H1.**  *Group efficacy is positively related to group performance.*

### 2.2. The Moderating Effect of Group Competency

Despite theoretical and empirical support for the positive relationship between group efficacy and group performance, some scholars have noted that it is a complex relationship contingent on factors that influence interpersonal interactions among group members [4,5,6,8,9,27]. Moreover, some empirical evidence suggests an opposite relationship between group efficacy and group performance. Hence, an excessively high level of group efficacy impairs group decision-making because members tend to take risks, reject negative feedback, and process information less vigilantly [28,29]. Rapp et al. (2014) found an inverted U-shaped relationship between group efficacy and group performance. Such paradoxical theoretical and empirical findings suggest that the relationship between group efficacy and group performance may differ depending on the context. For instance, research has shown that this relationship varies according to task characteristics [4,5,9], cultural context [6,9], and gender diversity [27]. 

Competency encompasses a set of individual behavioral characteristics that lead to career success in a specific organization [12,13,14]. Depending on the unique circumstances in which organizations find themselves, different employee characteristics are valued. For instance, organizations that pursue innovation may emphasize the proactivity and openness of employees, whereas those susceptible to serious safety hazards on the site would appreciate employee conscientiousness. Considering their idiosyncrasies, scholars and practitioners have highlighted the value of context-specific individual characteristics that directly contribute to an organization’s competitive advantage [30,31]. Competency reflects individuals’ potential and actual capabilities, which are not necessarily limited to behaviors considered desirable by the organization. Employees with high competency display the skills, attitudes, and behaviors that are considered effective in the organization [32]. In contrast, employees with low competency are perceived as incompetent workers and poor performers. Employee competency is not necessarily synonymous with performance, although they may be positively related in the long run. While one’s performance is influenced by external factors, such as support from supervisors, peers, and/or organizations, and the significance of tasks, competency is considered to be an inherent feature that may or may not be related to performance.

Competency differs from self-efficacy, performance, and competence [11]. First, while competency represents a set of individual characteristics, self-efficacy is a psychological state that may result from performance accomplishment, vicarious experiences, verbal persuasion, and emotional arousal. Indeed, Kurz and Bartram (2002) suggest that “competency is defined in relation to its significance for performance at work, rather than its content in purely psychological terms” (p. 231). Second, unlike competency which is inherently embedded in an individual, performance can be more susceptible to external factors other than an individual’s contribution and effort. Performance is usually rated based on job descriptions and the accomplishment of task goals, which are usually set according to a traditional task-based approach. Lastly, while competency includes a broad range of individual characteristics that are considered desirable in an organizational context, performance concerns achieving a predefined set of work-related standards.

Applying competency at the group level, the group average of competency indicates the group’s abilities and characteristics that are considered desirable in an organization. A low group average of competency indicates that the group lacks the capability to successfully achieve the group goal. We propose that the relationship between group efficacy and group performance, as rated by the group members, may vary according to the level of group average competency. Members with group efficacy commonly view group performance through a positive lens. This lenient tendency tends to intensify when there are members with deficits in their capabilities [33]. Kruger and Dunning (1999) proposed a state of “being ignorant of one’s own ignorance” [34]. Specifically, they suggest that those who lack knowledge and expertise make the choice they think is the most reasonable and believe in it, although it may not be the best choice in that situation. They do not know when they are mistaken or if others made a better choice. This tendency has been identified in people evaluating their own performance on exams [35,36], clinicians making mental illness diagnoses [37,38], physics experts who know which problems will be more difficult [39], and tennis players who know which shots are more likely to be winners [40]. Based on this rationale, we believe that the relationship between group efficacy and group performance rated by the members may differ according to the level of group competency. Specifically, we hypothesize that a group with a low level of competency tends to overestimate group performance driven by group efficacy, compared to a group with a high level of competency.

**H2.**  *Group average competency mitigates the relationship between group efficacy and group performance.*

Although research on leadership in teams [41] and exemplary members [42] provides evidence of the possible functional roles of individual members in improving group performance, little is known about the dysfunctional role of individual members, specifically that of incompetent ones. This missing piece is significant because social influence is the basic premise of social cognitive theory [2] To identify exemplary or dysfunctional roles, group composition literature has employed the general mental ability (GMA) construct. However, a meta-analysis [43] showed that distinctive group members’ GMA (i.e., the maximum and minimum) is not significantly related to group performance in field studies where researchers have little control over the context. Hence, GMA might not be the best indicator of group members’ abilities to perform their tasks within organizations, largely because of organizational idiosyncrasies. Thus, we employed competency, which reflects members’ organization-specific abilities and characteristics.

We argue that having an incompetent member may mitigate the positive effects of group efficacy on group performance. Although meta-analyses have demonstrated that group efficacy is often positively related to group performance [4,5], some empirical findings indicate that is not always the case [8,29]. Group efficacy is related to an expectancy or belief that a group can organize and execute the necessary actions to achieve its goal [2], leading to overconfidence and, in turn, failure to acknowledge weaknesses. In fact, the sense of confidence derived from group efficacy fosters excessive risk-taking, reduced attention to information processing, and inattention to negative feedback [29]. Similarly, groups of students with high group efficacy tend to perform worse than those with low group efficacy in coordinating individual activities in non-routine situations [44]. Even when a group has a bottleneck—an incompetent member slowing down the group process—an efficacious group will still set challenging goals and maintain aggressive strategies to achieve them. Consequently, it might not perform as well as a group that does not have a bottleneck.

Furthermore, social loafing literature suggests that the most incompetent member, who tends to be perceived as the poorest performer, may further undermine group productivity [20,45,46]. Social loafing refers to a voluntary reduction in motivation and work effort when individuals work in groups [47]. Research has identified antecedents, one of which is the perception of other members’ efforts [48]. Jackson and Harkins (1985) propose that team members tend to match their coworkers’ contributions. When a member is perceived as having a questionable work ethic and exhibiting behaviors indicating social loafing, other members may believe that this member is taking advantage of the group, thereby reducing their work efforts to avoid being taken for “suckers” [45,49]. We argue that the least competent member’s competency may cause the member to be perceived as a social loafer and lower the remaining members’ work motivation, driven by group efficacy. When the least competent member’s competency is low, other members may question their contribution and reduce their own work effort. In contrast, when the least competent member’s competency is still reasonably high, the remaining group members are less likely to feel that the member is a “free rider” and withhold effort. As such, we hypothesize a moderating effect of group minimum competency, operationalized as the least competent member’s competency, on the relationship between group efficacy and group performance.

**H3.**  *The competency of the least competent member within a group weakens the relationship between group efficacy and group performance.*

## 3. Methods

### 3.1. Sample and Procedure

As part of a company-wide consulting project, data were collected from employees of different departments, including finance, accounting, research and development, human resource management, management, sales, operational management, and supply management, at a manufacturing company in South Korea. The second author acquired the company’s archival data including group performance ratings and employees’ competency, and distributed additional paper and pencil surveys to measure the variables of interest including group efficacy and group performance rated by the group members. To avoid common method bias [50], data were collected from multiple sources in two waves. First, team leaders rated each member’s competency; after five months, the members rated group efficacy and group performance. After excluding participants who had left the company or entire teams that had dissolved during these two waves, 389 employees from 41 intact work groups were included in the analysis. Of the final respondents, 97% were male and the average age was 32 years. The summary of our sample statistics is presented in Table 1.

### 3.2. Measures

#### 3.2.1. Competency

Competency refers to employees’ abilities and characteristics that are considered desirable in a specific organizational context [11]. Employee competency was rated by their immediate supervisors using a five-item measure on a 5-point Likert scale. This measure was developed based on Boyatzis’s (1982) [51] competency model as a company-wide project. To develop the measure, the company conducted (1) content analysis by analyzing the vision statement and annual strategy report; (2) a workshop to define competent employees in the company; and (3) focus group interviews held by subject-matter experts to identify the generic characteristics of these competent employees, including conscientiousness, declarative skills and knowledge, understanding organizational culture, leadership, and managerial skills. Questions describing each characteristic were used. The least competent member had the lowest competency score within the group. We calculated inter-rater agreement (R_wg_) [52] to justify aggregation as needed (R_wg_ = 0.98). Studies have used 0.7 as a threshold to justify aggregating data across raters. Group average competency is operationalized as the level of competency of the group in general, and group minimum competency, operationalized as the least competent member’s competency.

#### 3.2.2. Group Efficacy

Group efficacy is defined as “a group’s shared belief in its conjoint capabilities to organize and execute the courses of action required to produce given levels of attainments” [2] (p. 477). Following Badura (1997) [2] and Gibson et al. (2000) [53], we aggregated each member’s appraisal of the group’s capabilities. Each member responded to the seven items developed by Riggs and Knight (1994) [54] on a seven-point Likert scale. A sample item is “The members of this department have excellent job skills.” Cronbach’s alpha was 0.83, and R_wg_ was 0.92.

#### 3.2.3. Group Performance

Group performance defined as the completion and the quality of the group task. Group performance was rated by Brannick et al. (1997) [55]. Sample items were “Our team’s work is high quality” and “So far, our team has been a great success.” Cronbach’s alpha for the scale was 0.85, and R_wg_ was 0.87.

#### 3.2.4. Control Variables

We controlled for the previous year’s group performance, group size, and department dummy, following previous studies. The previous year’s group performance is the results of the formal performance evaluation of upper-level supervisors. The performance evaluations range from 0 point to 100 points.

### 3.3. Data Analysis

Before testing our hypotheses, we verified the factor structure of group efficacy and group performance to ensure discriminant validity. We conducted Kaiser-Meyer-Olkin (KMO) test for sampling adequacy. The results show that KMO value was 0.88, which is greater than 0.60. Moreover, we also conducted Bartlett’s test of sphericity (χ^2^(55) = 1809.342, *p* < 0.000). Based on these results, we justified our principal component analysis. The results of the factor analysis is presented in Appendix A.

To test our hypotheses, a hierarchical regression analysis was conducted using SPSS software (version 27, IBM, Armonk, NY, USA). Given that the variables of interest were rated using different scales and issues of multicollinearity, they were standardized. We included control variables in Model 1 and then added the group efficacy, the independent variable in Model 2. In Model 3, we included moderators, the group competency. Finally, in Model 4 we tested the interaction effect of group efficacy and group competency. R^2^ of the models were calculated to show how much variance of group performance each model explains.

## 4. Results

Table 2 presents the means, standard deviations, and correlations between the variables of interest.

**Figure 2:**
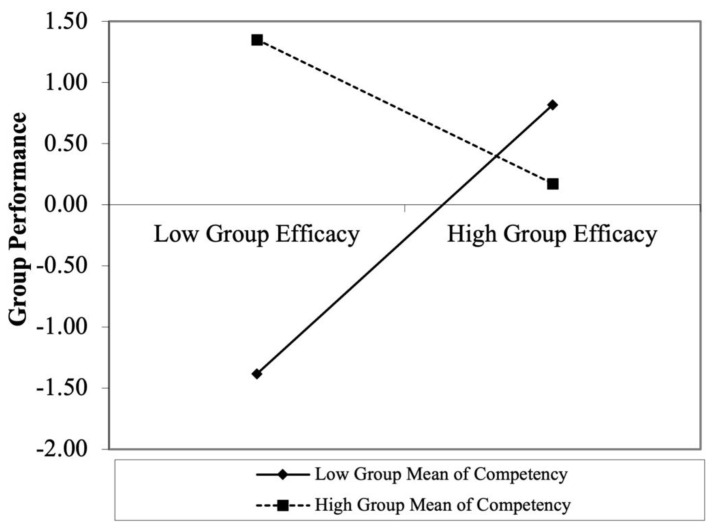
Moderating effect of group average competency on the relationship between group efficacy and performance.

**Figure 3:**
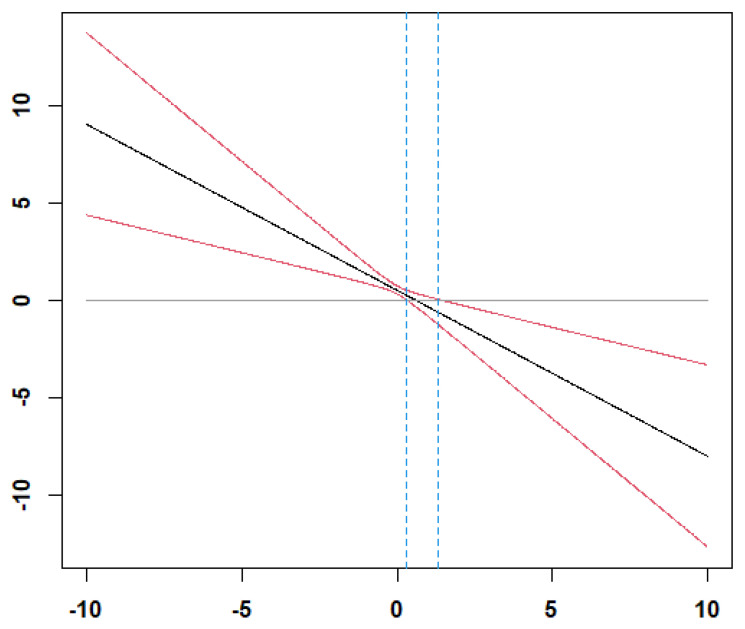
The region of significance of the moderating effect of group average competency. Note: The moderating effect is significant when the moderator is greater than 1.33 and smaller than 0.29. X-axis: Moderator, Y-axis: Simple slope.

The results presented in Table 2 show that group efficacy is positively related to group performance (β = 0.0.44, *p* < 0.05). Thus, Hypothesis 1 is supported. The results of Model 4 in Table 2 show that the interaction between group efficacy and group average competency is significantly related to group performance (β= −0.74, *p* < 0.05). Figure 2 illustrates the interaction effect when group average competency is low (1 SD below the mean) and group competency is high (1 SD above the mean). To further elucidate the significance of the moderating effect based on the levels of group competency, we illustrated the region of significance using the Johnson–Neyman technique [56]. Figure 3 shows that the moderating effect is significant when group average competency is greater than 1.33 and smaller than 0.29. Thus, the positive relationship between group efficacy and group performance is stronger when the group average is low than high. Thus, Hypothesis 2 is supported.

**Figure 4:**
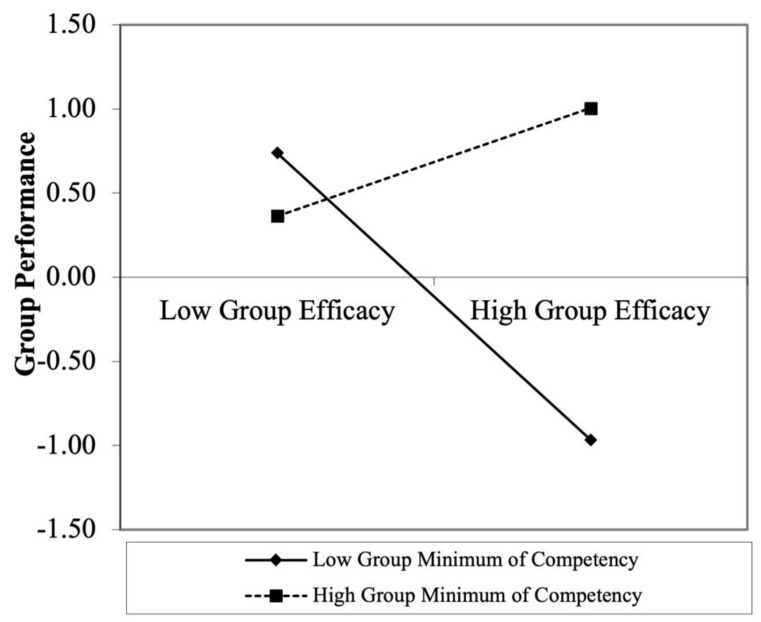
Moderating effect of group minimum competency on the relationship between group efficacy and performance.

**Figure 5:**
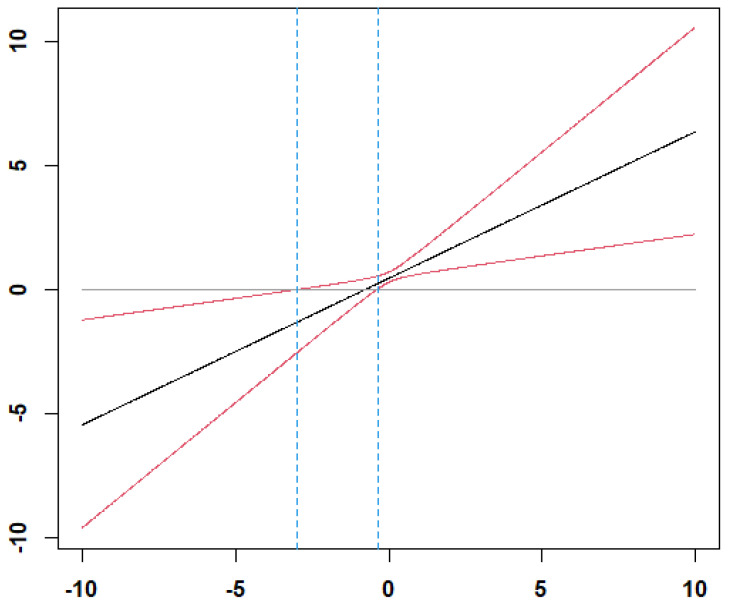
The region of significance of the moderating effect of group minimum competency. Note: The moderating effect is significant when the moderator is greater than −0.38 and smaller than −2.99. X-axis: Moderator, Y-axis: Simple slope.

The moderating effect of group minimum competency on the relationship between group efficacy and group performance is shown in Model 4 in Table 3 (β = 0.55, *p* < 0.05). The results in Figure 4 show that group efficacy deteriorates the quality of group performance when the competency of the least competent member in the group is low; however, the positive relationship remains when the competency of the least competent member in the group is high. The region of significance in Figure 5 shows that this moderating effect is significant when the moderator is greater than −0.38 and smaller than −2.99.

## 5. Discussion and Conclusions

We examined the moderating effects of group average and minimum competency on the relationship between group efficacy and performance. In concurrence with social cognitive theory [2] and prior meta-analytic findings [4,5], the positive relationship between group efficacy and group performance was verified. The more efficacious groups are, the higher they rate their own performance. This result is not surprising based on the prior research. We further found that the association between group efficacy and group performance varied according to the level of group competency. First, we found that group members with low levels of competency tended to overestimate their group performance as a result of group efficacy. This result provides additional support for the Dunning-Kruger effect [34]. That is, when members are incompetent, they tend to hold an overly optimistic view of their performance. Second, group efficacy lowers group performance when the competency of the least competent member is remarkably low. This result may seem to contradict social cognitive theory [2] yet supports the idea that a negative relationship may exist. According to perceptual control theory [57,58], a discrepancy between individuals’ current state and their desired state drives their motivation. From this perspective, one’s efficacy beliefs, which form an optimistic view of one’s current state, decrease the gap between current and desired states. Consequently, individuals with high self-efficacy invest fewer resources (e.g., time and effort) into completing their tasks, and performance quality tends to be compromised. Similarly, self-efficacy leads to overconfidence, inhibiting individuals from exerting effort [59] and therefore negatively affecting their performance [60,61]. In this vein, we propose a condition in which group efficacy may backfire. Although group efficacy encourages members to set and pursue challenging goals and presumably motivates members, groups with a bottleneck may not be able to coordinate the collective activities needed to achieve the group’s goals. Furthermore, groups may perform even worse if an incompetent member’s presence compromises other members’ motivation [49,62]. However, when the least competent member in the group still performs reasonably well, their presence is not as problematic.

### 5.1. Theoretical Implications

This study improves our understanding of such groups. First, we propose a moderating effect on the theoretically supported relationship between group efficacy and performance. Specifically, our results illustrate the group phenomenon, in which group efficacy may interfere with the group performance process in a context. Second, the results regarding the effects of the group average characteristic and least competent member provide insight into how groups function. While a rich body of research indicates that members’ characteristics are relevant to group performance [43], it mostly relies on the group mean or median and assumes that each member’s characteristics contribute equally to the collective pool of group characteristics. While this approach provides a general estimate of the group, a more nuanced approach that reveals how a unique member affects the group’s work has rarely been employed [18,63]. By illustrating how an incompetent member hampers a group’s functioning, the current study enhances our understanding of how to manage a work group.

### 5.2. Practical Implications

Our study provides insights on how to manage work teams. First, the possibility that group competency may influence the relationship between group efficacy and group performance should be considered when building and managing work groups. Second, managers should pay particular attention to incompetent members who may hamper group performance. While the ultimate goal of human resources personnel has been the recruitment and selection of competent employees, our results suggest that it is also important to dismiss or manage incompetent employees properly. Providing constructive feedback or training opportunities to incompetent employees is essential to improving group performance. Third, managers should be aware of the potential negative consequences of group efficacy, and how to cope with them. We found that the efficacy belief shared by members is related to group performance only when it is rated by group members, whereas it has a non-significant effect on the objective ratings of performance by upper management. This result indicates that efficacy beliefs do not improve group performance. In addition, we found that group efficacy is particularly detrimental when a group has a bottleneck; thus, rather than merely focusing on improving group efficacy, managers should also consider each member’s competency when designing work teams.

### 5.3. Limitations and Future Research

First, the generalizability of our results may be limited because our research model was tested using organization-specific information, such as the company’s measure of competency based on the general competency model [52], and the group performance ratings based on organization-specific criteria. Second, the mechanisms that explain the relationship between group efficacy and performance need to be investigated. Although our theoretical reasoning, based on social cognitive theory, suggests that group efficacy promotes members’ work motivation (which determines group performance), we did not measure group members’ motivation. Future studies should explore the mediators of the relationship between group efficacy and performance and how they interact with the minimum competency of the group. Additionally, future studies could investigate a more nuanced process through which the least competent member influences the group. For instance, social loafing could explain why bottlenecks affect the relationship between group efficacy and performance, and/or directly influence group efficacy. Jackson and Harkins (1985) [48] suggested that people are apt to match their work efforts to their coworkers when working together in a group. That is, social loafing in a group is likely to occur when a member is perceived as “free riding.” Third, this study did not consider task characteristics. Steiner (1972) [63] suggests that different configurations of group members’ abilities determine different types of group performance. Specifically, the group’s minimum of competency is crucial for conjunctive tasks, where a bottleneck slows down the entire process. Future studies may provide a more detailed analysis of the dynamics of how group members work together using Steiner’s (1972) [63] taxonomy of group tasks. Fourth, group performance from different sources may be employed to verify the hypothesized relationships. Although our study depicts the influence of group efficacy on member-rated group performance, it would be intriguing to examine the influence on group performance from different sources (e.g., objective measures and outsiders with relevant expertise). Finally, future study may take a closer look at the mechanisms that show in our finding. For instance, in addition to group efficacy, individual member’s self-efficacy could to also incorporated in the model [64]. Moreover, exploring antecedents of group efficacy may enrich our findings [65].

### 5.4. Conclusions

Although previous studies have supported the positive relationship between group efficacy and performance, some scholars have documented alternative relationships. Our study suggests that group competency is a moderator of the relationship between group efficacy and performance. Specifically, group average competency weakens the relationship between group efficacy and performance, implying that members tend to hold an optimistic view of their performance when their capabilities are limited. Moreover, we found that a bottleneck in an efficacious group represents at least one condition that forms a negative relationship between group efficacy and group performance.

## Figures and Tables

**Table 1:** Sample statistics.

Variables	Number	%
**Gender**		
**Female**	10	3.1
**Male**	314	96.9
**Missing value**	65	
**Age**		
**20~29**	132	39.6
**30~39**	176	52.9
**40 and above**	25	7.5
**Missing value**	56	
**Position**		
**Staff**	238	61.2
**Manager**	128	32.9
**Missing value**	23	
**Department**		
**Clerical**	97	24.9
**Non-clerical**	292	75.1

**Table 2:** Means, Standard Deviations, and Correlations among the Study Variables.

Variables	M	SD	1	2	3	4	5	6
1. Previous year’s group performance ^a^	75.59	11.20						
2. Department	0.54	0.51	−0.130					
3. Mean of competency	4.00	0.13	−0.063	−0.041				
4. Group size	9.51	5.89	0.069	0.242	−0.001			
5. Group efficacy	5.77	0.38	0.255	−0.305	0.045	−0.118		
6. Minimum of competency	3.62	0.32	−0.026	−0.142	0.749 **	−0.277	0.197	
7. Group performance ^b^	5.24	0.65	0.635 **	−0.166	−0.065	−0.060	0.515 **	0.064

Note: ^a^ Group performance rated by upper-level management supervisors. ^b^ Group performance rated by the group members. ** *p* < 0.01.

**Table 3:** Results of the Relationship between Group Efficacy, Competency, and Member-Rated Group Performance.

Variables	Model 1	Model 2	Model 3	Model 4
Step 1: Controls				
Previous year’s GP	0.591 **	0.486 **	0.473 **	0.561 **
Group size	0.031	0.083	0.026	−0.044
Department	−0.332 *	−0.240	−0.284	−0.490 **
Step 2: Main effect				
Group efficacy		0.440 **	0.505 **	0.517 **
Minimum of competency			−0.290	−0.299
Mean of competency			0.170	0.270
Step 3: Moderating effect				
GE × Mean of competency				−0.738 **
GE × Minimum of competency				0.551 **
Overall F	8.500 **	12.240 **	8.346 **	11.122 **
R^2^	0.505	0.671	0.695	0.816
F change	8.500 **	12.129 *	0.855	6.631 *
R^2^ change	0.505	0.166	0.024	0.122

Note. GP: Group Performance, GE: Group Efficacy, * *p* < 0.05, ** *p* < 0.001.

## Data Availability

Not applicable.

## Appendix A

**Table A1:** Survey Items for Group Efficacy and Group Performance and Factor Analysis Results.

	ITEMS	1	2
Group performance	1. Our team’s work is high quality.	0.268	**0.834**
	2. We are meeting our team objectives.	0.292	**0.739**
	3. Reports on our performance are favorable.	0.199	**0.862**
	4. So far, our team has been a great success.	0.137	**0.786**
Group efficacy	1. The department I work with has above average ability.	**0.626**	0.261
	2. (R) This department is poor compared to other departments doing similar work.	**0.704**	0.143
	3. (R) This department is not able to perform as well as it should.	**0.646**	0.350
	4. The members of this department have excellent job skills.	**0.706**	0.258
	5. (R) Some members of this department should be fired due to lack of ability.	**0.611**	0.055
	6. (R) This department is not very effective.	**0.681**	0.363
	7. (R) Some members in this department cannot do their jobs well.	**0.740**	0.141
	Eigen value	5.005	1.434
	Variance	45.504	13.032
